# The hidden cost of using low-resolution concentration data in the estimation of NH_3_ dry deposition fluxes

**DOI:** 10.1038/s41598-017-18021-6

**Published:** 2018-01-17

**Authors:** Frederik Schrader, Martijn Schaap, Undine Zöll, Richard Kranenburg, Christian Brümmer

**Affiliations:** 1 Thünen Institute of Climate-Smart Agriculture, Braunschweig, DE-38116 Germany; 2 TNO, Department of Climate, Air and Sustainability, Utrecht, NL-3584 The Netherlands

## Abstract

Long-term monitoring stations for atmospheric pollutants are often equipped with low-resolution concentration samplers. In this study, we analyse the errors associated with using monthly average ammonia concentrations as input variables for bidirectional biosphere-atmosphere exchange models, which are commonly used to estimate dry deposition fluxes. Previous studies often failed to account for a potential correlation between ammonia exchange velocities and ambient concentrations. We formally derive the exact magnitude of these errors from statistical considerations and propose a correction scheme based on parallel measurements using high-frequency analysers. In case studies using both modelled and measured ammonia concentrations and micrometeorological drivers from sites with varying pollution levels, we were able to substantially reduce bias in the predicted ammonia fluxes. Neglecting to account for these errors can, in some cases, lead to significantly biased deposition estimates compared to using high-frequency instrumentation or corrected averaging strategies. Our study presents a first step towards a unified correction scheme for data from nation-wide air pollutant monitoring networks to be used in chemical transport and air quality models.

## Introduction

Gaseous ammonia (NH_3_) plays an important role in the atmosphere as part of the natural and anthropogenic N cycle and contributes to a number of adverse effects on the environment and public health^1^. Recent developments allow the direct quantification of NH_3_ dry deposition and emission fluxes via the eddy-covariance method^2–4^; however, the necessary instrumentation is costly, long-term continuous studies are yet to be published, and the method is not trivially applicable in every environment. Alternative methods, such as the aerodynamic gradient technique, are even more labour-intensive, usually require expensive wet-chemical analyses, and are prone to errors in non-ideal conditions^5^.

A cost- and labour-efficient alternative to flux measurements is the use of so-called dry deposition inferential models. If they are properly validated against flux measurements in different ecosystems, they can be applied for regional estimates of NH_3_ dry deposition using only concentration measurements and a small number of (micro-)meteorological variables as input data^6–10^. These models are usually ran on a 30 minute basis, in accordance with the typical temporal resolution of flux measurements, or on an hourly basis within some large-scale chemistry transport models (CTM), such as LOTOS-EUROS^11,12^. However, in national monitoring networks, such as the Measuring Ammonia in Nature (MAN) network in the Netherlands^13^, often passive samplers or denuders (e.g. DELTA^14^, or KAPS^15,16^) are used to measure ambient NH_3_ concentrations, which typically only yield a temporal resolution of monthly averages. The impact of using such low-resolution concentration measurements as input data for bidirectional NH_3_ dry deposition inferential models has, to our knowledge, not been thoroughly investigated in the published literature, although they have regularly been used from local studies^17,18^ to integrated projects^19^. In order to systematically assess potential bias introduced by using low-resolution concentration data, we exemplarily analysed a 1 year gap-free record of ambient NH_3_ concentrations predicted by the CTM LOTOS-EUROS in conjunction with ECMWF (European Centre for Medium-Range Weather Forecasts) meteorology as input data for an independent dry deposition inferential model by Massad *et al*.^7^, as well as preliminary NH_3_ concentration measurements using quantum cascade laser (QCL) spectroscopy at a remote site in Germany. We investigated the potential magnitude of errors introduced by using low-resolution concentration measurements and formally derived the fundamental equations necessary for the development of correction schemes. Our study lays the groundwork for the characterisation of errors and estimation of site-specific correction functions when using NH_3_ dry deposition models with low-resolution input data.

## Methods

### Dry deposition inferential modelling

**Figure 1:**
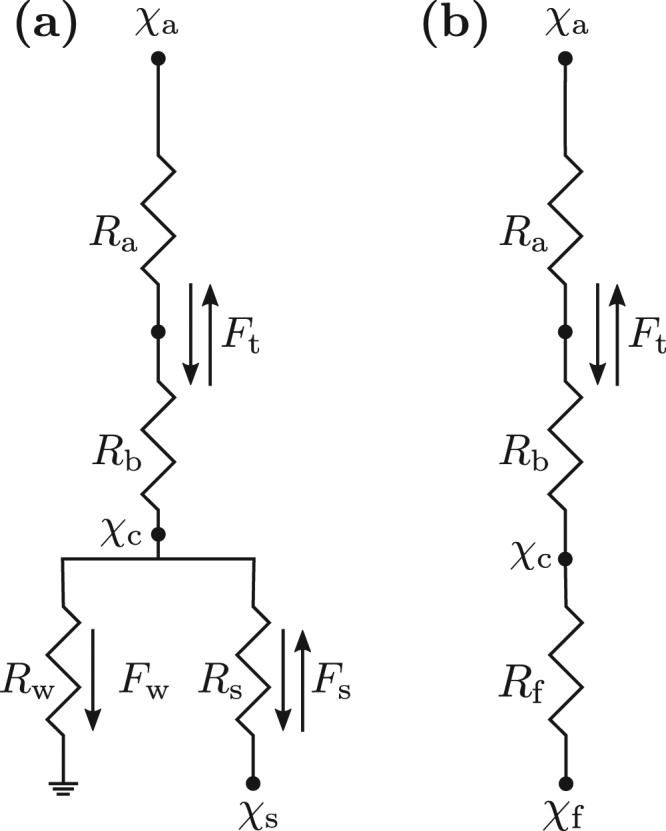
(**a**) Common structure of a bidirectional one-layer canopy compensation point model for biosphere-atmosphere exchange of NH_3_. (**b**) Simplification of (**a**) to a serial resistance structure.

Dry deposition of NH_3_ is most commonly modelled using parameterisations of a big-leaf canopy compensation point model, or a two-layer variant thereof when exchange with the soil- or litter-layer is expected to be significant and can be parameterised within reasonable margins of uncertainty^6,7,20^. We here use the parameterisation of Massad *et al*.^7^ in a one-layer configuration to ensure independence from the dry deposition module (DEPAC within LOTOS-EUROS) involved in the generation of the synthetic data. In this model, the flux density of NH_3_ is predicted from the difference of the measured air NH_3_ concentration *χ*_a_ (μg NH_3_ m^−3^) at the aerodynamic reference height *z* − *d* (m) and the (modelled) canopy compensation point concentration, *χ*_c_ (μg NH_3_ m^−3^) (Fig. 1). The sign of this difference governs the direction of the flux (*χ*_a_ > *χ*_c_ leads to a deposition flux, with a negative sign by convention, and *χ*_a_ < *χ*_c_ leads to an emission flux). Furthermore, the magnitude of the predicted flux density is controlled by the magnitude (i) of *χ*_a_ − *χ*_c_, and (ii) of a number of resistances towards deposition. Within this framework, the total net biosphere-atmosphere exchange flux of NH_3_, *F* (μg NH_3_ m^−2^ s^−1^), is typically given as

1 $$F=-\frac{{\chi }_{{\rm{a}}}-{\chi }_{{\rm{c}}}}{{R}_{{\rm{a}}}+{R}_{{\rm{b}}}},$$

where *R*_a_ (s m^−1^) and *R*_b_ (s m^−1^) are the aerodynamic and quasi-laminar boundary layer resistance, respectively, and are here modelled as described in detail by Massad *et al*.^7^. Instead of calculating the canopy compensation point (which is a function of both stomatal and cuticular resistance and, if applicable, their respective compensation points, and the air NH_3_ concentration), we can simplify the model scheme to strictly consist of serial resistances only (Fig. 1b). The effective ‘foliar compensation point’, *χ*_f_ (μg NH_3_ m^−3^), is then given as a weighted average of both leaf-layer pathways via

2 $${\chi }_{{\rm{f}}}=\frac{{R}_{{\rm{f}}}}{{R}_{{\rm{w}}}}\cdot {\chi }_{{\rm{w}}}+\frac{{R}_{{\rm{f}}}}{{R}_{{\rm{s}}}}\cdot {\chi }_{{\rm{s}}},$$

where *χ*_w_ (μg NH_3_ m^−3^) and *χ*_s_ (μg NH_3_ m^−3^) are the cuticular and stomatal compensation point, respectively, *R*_w_ (s m^−1^) is the cuticular resistance, parameterised after Massad *et al*.^7^, and *R*_s_ (s m^−1^) is the stomatal resistance after Emberson *et al*.^21^. In the Massad *et al*.^7^ parameterisation, *χ*_w_ is zero (i.e., only deposition to the cuticula is possible). *R*_f_ (s m^−1^) is the ‘foliar resistance’, similar to the notation of Wichink Kruit *et al*.^8^, and is given as

3 $${R}_{{\rm{f}}}={({{R}_{{\rm{w}}}}^{-1}+{{R}_{{\rm{s}}}}^{-1})}^{-1}.$$

To further simplify the calculations, we define an exchange velocity, *v*_ex_ (m s^−1^), as the inverse of the total resistance to NH_3_ exchange:

4 $${v}_{{\rm{ex}}}={({R}_{{\rm{a}}}+{R}_{{\rm{b}}}+{R}_{{\rm{f}}})}^{-1}.$$

Note that *R*_f_ is not necessarily equal to the so-called canopy resistance, which is usually only used in unidirectional (deposition-only) models (i.e., they are only equal when *χ*_f_ is zero). Similarly, *v*_ex_ is not equal to the common concept of a deposition velocity, in which *R*_f_ is replaced by the canopy resistance and which is not used in conjunction with a compensation point.

Given these definitions, the net NH_3_ exchange flux can also be written as

5 $$\begin{array}{rcl}F & = & -{v}_{{\rm{ex}}}\cdot ({\chi }_{{\rm{a}}}-{\chi }_{{\rm{f}}})\\  & = & {v}_{{\rm{ex}}}\cdot ({\chi }_{{\rm{f}}}-{\chi }_{{\rm{a}}}),\end{array}$$

where a positive flux indicates emission and a negative flux indicates deposition.

### Flux prediction strategies for low-resolution input concentrations

High-frequency concentration measurements are often cost- and labour-intensive, and usually not available within nationwide long-term monitoring networks. A number of different variants to predict long-term average or cumulative flux densities from low-frequency concentration measurements can be found in the literature^18,19,22–28^. We here discuss the case of monthly averages, which are a common outcome of passive sampler or denuder measurements, but the calculations remain the same for any other kind of averaging period. A sensitivity study for other averaging periods is given in the Supplementary Material.

Consider the case of (i) (micro-)meteorological input data available at a sampling frequency the model is usually run at, e.g. 1 hour in our sample datasets, and (ii) ambient NH_3_ concentrations available at a lower sampling frequency, e.g. 1 month. We further assume that, from these data, reasonable flux predictions can only be made at the lowest available time scale, i.e. 1 month in this example. However, the model should still be run at a higher resolution in order to incorporate the effects of diurnal variations and day-to-day variability in meteorological conditions. There are generally two straightforward strategies to predict monthly averaged NH_3_ fluxes under these conditions, the first being:

6 $$\overline{F}=\overline{{v}_{{\rm{ex}}}\cdot ({\chi }_{{\rm{f}}}-\overline{{\chi }_{{\rm{a}}}})}$$

Here, an overbar $\overline{x}$ denotes the arithmetic mean of some random variable *x*, and a prime *x′* denotes the instantaneous deviation from $\overline{x}$, i.e. $x=\overline{x}+x^{\prime}$, similar to the notation commonly employed by the micrometeorological community. Consequently, equation (6) means that the model is run on an hourly basis with hourly meteorological input data, and the measured monthly average NH_3_ concentration is used as a substitute for hourly concentration values. In other words, it is assumed that the monthly average NH_3_ concentration is representative for hourly values and that internal mechanics of the model (such as the exponentially temperature dependent conversion of emission potentials to compensation points^20^) effectively compensate the effect of the lowered input data resolution.

An alternative strategy would be to first calculate the exchange velocity and the compensation points at a high resolution (given that they are independent of *χ*_a_), average them, and then calculate the monthly average flux from the monthly average of all other variables:

7 $$\overline{F}=\overline{{v}_{{\rm{ex}}}}\cdot (\overline{{\chi }_{{\rm{f}}}}-\overline{{\chi }_{{\rm{a}}}})$$

We will outline in the following section why both of these variants (equations (6) and (7)) will inevitably lead to biased results.

### Derivation of the error term

A well-understood, but still often ignored fallacy is the assumption that the product of averages yields similar results to the average of products^29^. However, this is generally only the case when all variables involved are completely independent and uncorrelated. Even if not all of these variables are formally linked within the governing equations of a dry deposition model, they may be correlated through their inherent dependence on external environmental factors (e.g. temperature, radiation, or turbulence). Meyers & Yuen^30^ were among the first to observe the impacts of ignoring this fallacy with regards to (unidirectional) inferential modelling of SO_2_ and O_3_ fluxes. For a bidirectional NH_3_ exchange scheme, the true mean flux over a certain period of time can be written as:

8 $$\begin{array}{ll}\overline{F} & =\,\overline{{v}_{{\rm{ex}}}\cdot ({\chi }_{{\rm{f}}}-{\chi }_{{\rm{a}}})}\\  & \ne \,\overline{{v}_{{\rm{ex}}}}\cdot (\overline{{\chi }_{{\rm{f}}}}-\overline{{\chi }_{{\rm{a}}}})\mathrm{.}\end{array}$$

Recall our definition of $\overline{x}$ and $x^{\prime}$, from which it follows that ${x^{\prime} }^{2}$ is equal to the (non Bessel-corrected) variance of *x*, and $\overline{x^{\prime} \cdot y^{\prime} }$ to the covariance of two random variables *x* and *y*. With these additional definitions, we can calculate the true average flux from long-term average NH_3_ concentrations using the linearity of expected values and the definition of the covariance, as follows:

9 $$\begin{array}{cc}\bar{F} & =\,\bar{{v}_{{\rm{e}}{\rm{x}}}\cdot ({\chi }_{{\rm{f}}}-{\chi }_{{\rm{a}}})}\\  & =\,\bar{{v}_{{\rm{e}}{\rm{x}}}\cdot {\chi }_{{\rm{f}}}}-\bar{{v}_{{\rm{e}}{\rm{x}}}\cdot {\chi }_{{\rm{a}}}}\\  & =\,\bar{{v}_{{\rm{e}}{\rm{x}}}}\cdot \bar{{\chi }_{{\rm{f}}}}+\overline{{v}_{{\rm{e}}{\rm{x}}}^{\prime} \cdot {\chi }_{{\rm{f}}}^{\prime} }-(\bar{{v}_{{\rm{e}}{\rm{x}}}}\cdot \bar{{\chi }_{{\rm{a}}}}+\overline{{v}_{{\rm{e}}{\rm{x}}}^{\prime} \cdot {\chi }_{{\rm{a}}}^{\prime} })\\  & =\,\bar{{v}_{{\rm{e}}{\rm{x}}}}\cdot (\bar{{\chi }_{{\rm{f}}}}-\bar{{\chi }_{{\rm{a}}}})+\overline{{v}_{{\rm{e}}{\rm{x}}}^{\prime} \cdot {\chi }_{{\rm{f}}}^{\prime} }-\overline{{v}_{{\rm{e}}{\rm{x}}}^{\prime} \cdot {\chi }_{{\rm{a}}}^{\prime} }.\end{array}$$

The difference between equation (7) and the last line of equation (9), i.e., the two covariance terms $\overline{{v}_{{\rm{e}}{\rm{x}}}^{\prime} \cdot {\chi }_{{\rm{f}}}^{\prime} }-\overline{{v}_{{\rm{e}}{\rm{x}}}^{\prime} \cdot {\chi }_{{\rm{a}}}^{\prime} }$ (μg NH_3_ m^−2^ s^−1^), is equal to the exact error introduced when calculating average NH_3_ fluxes from average exchange velocities and measured long-term average concentration measurements. When directly calculating hourly fluxes with the long-term average NH_3_ concentrations used as a substitute for hourly values and averaging afterwards (i.e., using equation (6)), the error is equal to $-\overline{{v}_{{\rm{e}}{\rm{x}}}^{\prime} \cdot {\chi }_{{\rm{a}}}^{\prime} }$ (μg NH_3_ m^−2^ s^−1^).

### A first step towards bias elimination

If we run a dry deposition inferential model with only the ambient NH_3_ concentration as a long-term average and all other driving variables measured at a higher temporal resolution, as is usually the case for monitoring stations, where the measurement of meteorological variables at a high temporal resolution is not very difficult, only the last term of equation (9), i.e. the covariance of *v*_ex_ and *χ*_a_, is unknown. We can expand it to take the form

10 $$\overline{{v}_{{\rm{e}}{\rm{x}}}^{\prime} \cdot {\chi }_{{\rm{a}}}^{\prime} }=\sqrt{\bar{{{v}_{{\rm{e}}{\rm{x}}}^{\prime} }^{2}}}\cdot \sqrt{\bar{{{\chi }_{{\rm{a}}}^{\prime} }^{2}}}\cdot \frac{\overline{{v}_{{\rm{e}}{\rm{x}}}^{\prime} \cdot {\chi }_{{\rm{a}}}^{\prime} }}{\sqrt{\bar{{{v}_{{\rm{e}}{\rm{x}}}^{\prime} }^{2}}}\cdot \sqrt{\bar{{{\chi }_{{\rm{a}}}^{\prime} }^{2}}}}.$$

Note that here $\sqrt{\overline{{{v}_{{\rm{ex}}}^{\prime} }^{2}}}={\sigma }_{{v}_{{\rm{ex}}}}$ (m s^−1^) and $\sqrt{\overline{{{\chi }_{{\rm{a}}}^{\prime} }^{2}}}={\sigma }_{{\chi }_{{\rm{a}}}}$ (μg NH_3_ m^−3^) are identical to the empirical standard deviation of *v*_ex_ and *χ*_a_, respectively, and $\overline{{v}_{{\rm{e}}{\rm{x}}}^{\prime} \cdot {\chi }_{{\rm{a}}}^{\prime} }\cdot {(\sqrt{\bar{{{v}_{{\rm{e}}{\rm{x}}}^{\prime} }^{2}}}\cdot \sqrt{\bar{{{\chi }_{{\rm{a}}}^{\prime} }^{2}}})}^{-1}={r}_{{v}_{{\rm{e}}{\rm{x}}},{\chi }_{{\rm{a}}}}$ (−) is equal to the Pearson product-moment correlation of the two. Again, ${\sigma }_{{v}_{{\rm{ex}}}}$ is known and can trivially be calculated from higher-resolution modelled estimates of *v*_ex_. However, ${\sigma }_{{\chi }_{{\rm{a}}}}$ and ${r}_{{v}_{{\rm{ex}}},{\chi }_{{\rm{a}}}}$ remain unknown at this point.

A simple approach to calculate less-biased fluxes from low-resolution concentration measurements could be based on accompanying high-resolution concentration measurements at the same site for a limited amount of time. E.g., one would use a single ‘high-frequency’ (0.5 to 1 hour sampling rate) NH_3_ monitor to take parallel measurements at a monitoring site for a few months to gather the necessary data to derive correction factors, and then move the instrument to the next site. We can assume an increase of the variation in air NH_3_ concentrations with rising concentration levels, i.e. increasing ${\sigma }_{{\chi }_{{\rm{a}}}}$, with increasing mean $\overline{{\chi }_{{\rm{a}}}}$, since (i) chemical measurement instruments often exhibit relative errors, and (ii) it is reasonable to suspect that, for instance, emissions from nearby sources would not only lead to a steady increase of the mean NH_3_ concentration, but also to a higher variability, depending on turbulent mixing, wind direction, and other factors. The most simple approach is to model this with a linear relationship:

11 $${\hat{\sigma }}_{{\chi }_{{\rm{a}}}}(\overline{{\chi }_{{\rm{a}}}})={m}_{0}\cdot \overline{{\chi }_{{\rm{a}}}}+{b}_{0},$$

where *m*_0_ (−) and *b*_0_ (μg NH_3_ m^−3^) are the slope and intercept of the resulting regression line, respectively.

Modelling the correlation between the exchange velocity and the air NH_3_ concentration, ${r}_{{v}_{{\rm{ex}}},{\chi }_{{\rm{a}}}}$, is substantially less straightforward. A zeroth-order approach would consist of simply taking the mean correlation over the measurement period used for deriving correction functions. However, this would eliminate the possibility of registering potential seasonality in ${r}_{{v}_{{\rm{ex}}},{\chi }_{{\rm{a}}}}$, and the next most simple alternative, a linear regression of ${r}_{{v}_{{\rm{ex}}},{\chi }_{{\rm{a}}}}$ against some environmental variable, would yield practically the same results if the slope of the regression is close to zero, leaving little reason not to favour at least a simple linear regression over the mean. Unfortunately, the choice of a suitable explanatory variable is far from trivial, as we essentially look for a correlation of a correlation, which is a somewhat ill-defined and difficult to understand concept. We will here exemplarily perform a linear regression against temperature ${T}_{\mathrm{air}}(^\circ {\rm{C}})$, assuming that with rising temperature (as a measure for the energy content of the system), both volatilisation of NH_3_ and buoyancy will increase and the corr\elation between the two might become stronger. However, this is merely an educated guess and not bound to be the most suitable model, nor is temperature guaranteed the most suitable regressor. In fact, we suspect that especially at remote sites with little to no diurnal variation in air NH_3_ concentrations, but pronounced variation in *v*_ex_ (which is strongly linked to atmospheric turbulence), most variables with a strong diurnal cycle would work similarly well as a predictor for ${r}_{{v}_{{\rm{ex}}},{\chi }_{{\rm{a}}}}$. The model is given as:

12 $${\hat{r}}_{{v}_{{\rm{e}}{\rm{x}}},{\chi }_{{\rm{a}}}}(\bar{{T}_{{\rm{a}}{\rm{i}}{\rm{r}}}})={m}_{1}\cdot \bar{{T}_{{\rm{a}}{\rm{i}}{\rm{r}}}}+{b}_{1},$$

with the slope *m*_1_ (−) and intercept *b*_1_ (−). Equation (9) then becomes:

13 $$\bar{F}=\bar{{v}_{{\rm{e}}{\rm{x}}}}\cdot (\bar{{\chi }_{{\rm{f}}}}-\bar{{\chi }_{{\rm{a}}}})+\bar{{v}_{{\rm{e}}{\rm{x}}}^{\prime} \cdot {\chi }_{{\rm{f}}}^{\prime} }-{\sigma }_{{v}_{{\rm{e}}{\rm{x}}}}\cdot {\hat{\sigma }}_{{\chi }_{{\rm{a}}}}(\bar{{\chi }_{{\rm{a}}}})\cdot {\hat{r}}_{{v}_{{\rm{e}}{\rm{x}}},{\chi }_{{\rm{a}}}}(\bar{{T}_{{\rm{a}}{\rm{i}}{\rm{r}}}}).$$

### Comparison of flux prediction strategies

A 12-month gap-free set of ‘synthetic’ input data was generated by running the Eulerian grid model LOTOS-EUROS^31^ in conjunction with ECMWF meteorology for the year 2016. Through a one-way nesting procedure a simulation over Germany was performed on a resolution of 0.125° longitude by 0.0625° latitude, approximately 7 by 7 km^2^. The high resolution domain is nested in a European domain with a resolution of 0.5° longitude by 0.25° latitude, approximately 28 by 28 km^2^. Emissions include the TNO MACC-III European emission inventory for the year 2014. For Germany, the national emission inventory of the German Environmental Protection Agency (UBA) was used to prescribe the gridded emissions. LOTOS-EUROS is one of the few CTMs that include SO_2_-NH_3_ co-deposition and bidirectional surface-atmosphere exchange of NH_3_^12,32^.

**Figure 2:**
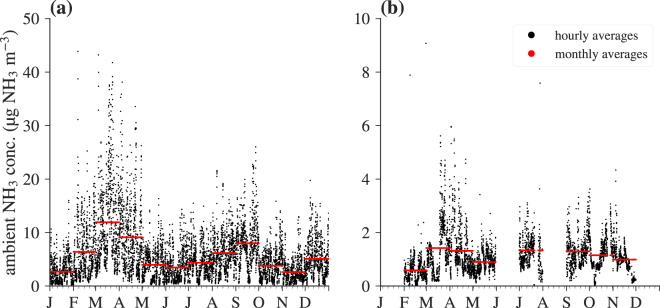
Hourly and monthly averaged air NH_3_ concentrations for the year 2016 of (**a**) synthetic data predicted from LOTOS-EUROS for one grid cell in the Allgäu region in Germany and (**b**) measured data from a flux tower in the Bavarian Forest.

We here used data from one grid cell in the Allgäu region in southern Germany (47°41′34.80′′ N, 10°2′6.00′′ E) (Fig. 2a). Average temperature during the year of 2016 was 8.1 °C, total precipitation 1690 mm, and the average NH_3_ concentration was 5.6 μg NH_3_ m^−3^ (highest hourly means up to 60.6 μg NH_3_ m^−3^) at an (aerodynamic) reference height of 2.5 m above zero-plane displacement. The annual course of the leaf area index was modelled as implemented in the DEPAC deposition module within LOTOS-EUROS^12,21^. We here exemplarily used land-use parameters for grassy semi-natural vegetation; results for other land-use classes can be found in Fig. S1 of the Supplementary Material.

Additionally, we tested the correction scheme for measured data from a flux tower in the Bavarian Forest in Germany (Fig. 2b) at 807 m a.s.l. (base of the tower), 48°56′50.27′′ N, 13°25′12.22′′ E^33^. NH_3_ concentrations were measured using a QCL absorption spectrometer from Aerodyne Research Inc., Billerica, MA, USA (cf. Zöll *et al*.^2^. for a detailed instrument description) at 31 m above ground level, and with an original sampling rate of 10 Hz averaged to 1 concentration value per hour. Turbulence measurements were taken with a sonic anemometer (model R3, Gill Instruments Ltd., Lymington, UK) at the same height, as well as temperature and relative humidity using HC2S3 probes (Campbell Scientific, Inc., Logan, UT, USA). Leaf area index and canopy height were not measured at the site and parameterised as proposed in Massad *et al*.^7^. Annual average temperature at the site was 7.4 °C, total precipitation was 1047 mm, and the average of NH_3_ concentrations used in this study (approximately 56% data coverage of the year) was 1.1 μg NH_3_ m^−3^ (maximum 14.5 μg NH_3_ m^−3^). Measured ambient NH_3_ concentrations at this site are preliminary, but have undergone common quality procedures, such as despiking, and system performance tests with regard to flow rate, temperature, and pressure stability. These data will be published in an ecological context in the near future. The purpose of using this dataset is solely meant for assessing the correction scheme, thus absolute numbers should not be cited for verifying ecosystem-specific thresholds. We also note that we here used the Massad *et al*.^7^ parameterisation in its original form, despite the findings of Schrader *et al*.^34^ regarding a likely too large non-stomatal (cuticular) resistance in this parameterisation. While this leads to relatively low predicted fluxes, the derivation of the error term is unaffected. An additional case study for a moorland site in southern Scotland can be found in Fig. S2 in the Supplementary Material.

The dry deposition inferential model was run for four different scenarios for each site:

1. ‘control’: all variables at hourly resolution; flux calculation on hourly basis and subsequent averaging to monthly average fluxes (equation (8)).
2. ‘direct’: monthly average NH_3_ concentrations and all other variables at hourly resolution; flux calculation on hourly basis with hourly NH_3_ concentrations substituted by their monthly averages; subsequent averaging to monthly average fluxes (equation (6)).
3. ‘monthly’: monthly average NH_3_ concentrations and all other variables at hourly resolution; calculation of exchange velocities and foliar compensation points on hourly basis; subsequent averaging to monthly average exchange velocities and foliar compensation points, and calculation of monthly fluxes via equation (7).
4. ‘corrected’: same as ‘monthly’, but with added correction terms from equations (11)–(13).

Note that ‘corrected’ can also be written as equal to ‘direct’ plus only the correction term for the covariance of *v*_ex_ and *χ*_a_. Monthly deposition fluxes for months with gaps in the measured dataset were calculated by multiplying the arithmetic mean flux density of a given month with the number of data points at 100% coverage (assuming no bias of the gaps towards a certain time of the day).

### Code and data availability

Synthetic data are available at reasonable request from M. Schaap. Measured data will be published separately after final analysis. A Python 2.7 implementation of the Massad *et al*.^7^ parameterisation can be requested from the lead author. An open source version of LOTOS-EUROS is publicly available.

## Results and Discussion

**Figure 3:**
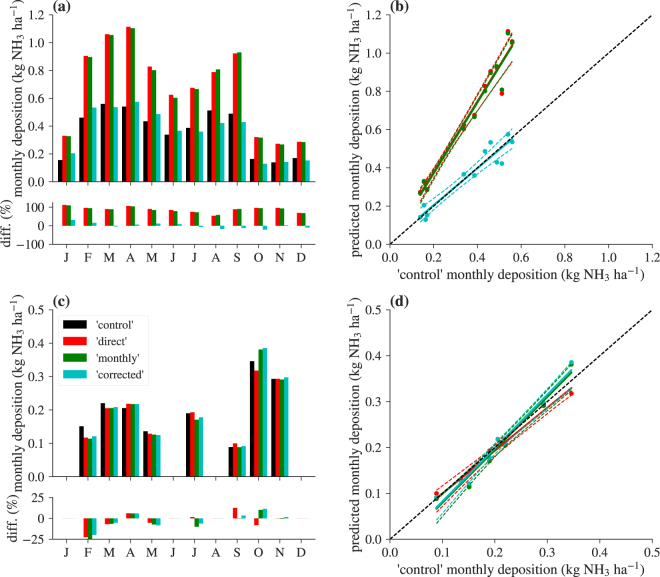
(**a**) Predicted cumulative monthly NH_3_ deposition for the four scenarios ‘control’, ‘direct’, ‘monthly’, and ‘corrected’ of the synthetic dataset (see text for description). Differences are given as percent deviation from ‘control’. (**b**) Predicted cumulative monthly NH_3_ deposition of ‘direct’, ‘monthly’, and ‘corrected’ variants against ‘control’. Dashed lines are 95% bootstrapped confidence intervals of the regression lines. (**c**,**d**) Same as (**a**,**b**), but for the measured data. The legend in (**c**) is valid for all four panels.

Figure 3a,b exemplarily shows the results of the comparison between the different averaging strategies for the synthetic dataset, using the parameterisation of the dry deposition model for semi-natural ecosystems. During some months, the relative error reaches over 100% higher predicted deposition compared to ‘control’ in a given month (e.g., January and April). The lowest error introduced by using uncorrected averaging strategies is in August (54% for the ‘direct’ variant, and 58% for the ‘monthly’ variant). Overall, the uncorrected variants overestimate total NH_3_ dry deposition for the year 2016 roughly by a factor of two (Table 1). There is no clear dependency of the magnitude of the relative error on environmental drivers apparent from our observations; however, the magnitude of the error is naturally strongly linked to ${r}_{{{\rm{v}}}_{{\rm{ex}}}{,{\rm{\chi }}}_{{\rm{a}}}}$. Consequently, the performance of a correction scheme is directly proportional to the certainty with which the correlation of the exchange velocity and the air NH_3_ concentration can be estimated. It also directly follows from a special case of equation (9), when *χ*_f_ is assumed to be zero, that the use of average deposition velocities instead of effective deposition velocities in a unidirectional framework is affected by the exact same type of error. In fact, due to the implicit integration of the compensation point in the deposition velocity, the error can be expected to be larger. Similar observations have been made by Matt & Meyers^35^ and Meyers & Yuen^30^ for SO_2_ and O_3_, in which they attempted to reduce the error by employing day- and night-sampling strategies. The proposed correction approach leads to a strong improvement during all months (Table 1, Fig. 3a,b), especially considering its relative simplicity. These findings are also confirmed by running the model for different synthetic datasets with different land-use types (Table S1 and Fig. S1 in the Supplementary Material).

**Table 1:** Performance of the different averaging strategies.

	Scenario	Coverage (%)	Σ*F* (g NH_3_ ha^−1^)	Difference (g NH_3_ ha^−1^)	MAD (g NH_3_ ha^−1^)
synthetic	‘control’	100	4347.0		
	‘direct’		8126.5	3779.5	315.0
	‘monthly’		8062.1	3715.0	309.6
	‘corrected’		4329.0	−18.0	40.9
measured	‘control’	56	1629.6		
	‘direct’		1572.8	−56.8	13.9
	‘monthly’		1594.5	−35.1	16.2
	‘corrected’		1624.7	−4.9	15.5

Coverage: Raw data coverage of the year 2016; Σ*F*: Sum of all monthly fluxes (positive is deposition); Difference: difference from ‘control’; MAD: mean absolute monthly differences from ‘control’.

**Figure 4:**
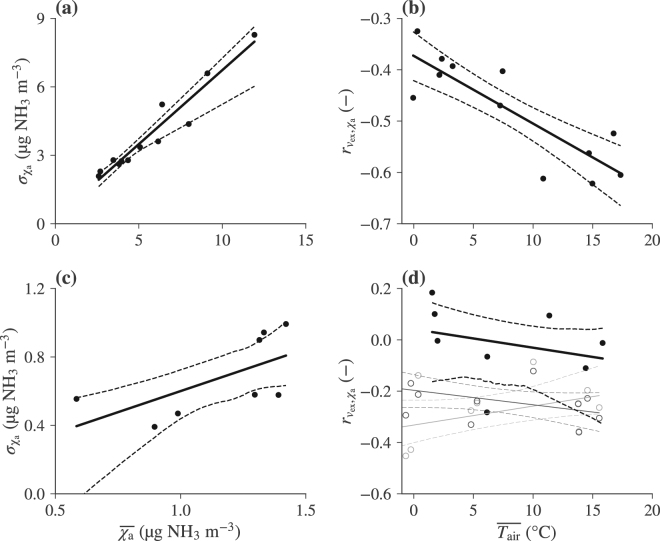
Linear regressions as an estimate for (**a**) the monthly standard deviation of air NH_3_ concentrations and (**b**) the monthly Pearson correlation of exchange velocities and air NH_3_ concentrations for the synthetic dataset. (**c**,**d**) Same as (**a**,**b**), but for the measured data. Grey lines in panel (**d**) are results for CTM data from the grid cell that includes the Bavarian Forest measurement tower. Light grey is modelled with land-use parameters for a coniferous, dark grey for a deciduous forest.

For the measured data (Fig. 3c,d), the picture is somewhat less clear, due to alternating over- and underestimations of averaged predicted deposition with respect to the control. While in some months, uncorrected ‘direct’ or ‘monthly’ flux prediction strategies give the best approximation to flux calculation using high-frequency data (e.g., May or October), the sum of all deviations from ‘control’ is still lowest for the ‘corrected’ variant (Table 1). However, the mean absolute deviation from ‘control’ is lowest for the ‘direct’ variant, albeit by a very small margin. Reasons for this less clear performance can be found in the very uncertain regression of ${r}_{{v}_{{\rm{ex}}},{\chi }_{{\rm{a}}}}$ against ambient temperature (Fig. 4d), leading to all estimates of ${r}_{{v}_{{\rm{ex}}},{\chi }_{{\rm{a}}}}$ close to its arithmetic mean, although it clearly changes throughout the measurement period. Furthermore, the error introduced by the different averaging strategies is already much lower (<25%) than for the synthetic dataset, which indicates a strong site-specificity of ${r}_{{v}_{{\rm{ex}}},{\chi }_{{\rm{a}}}}$. This is supported by the observation that other measured datasets exhibit much larger errors (Fig. S2 in the Supplement).

The higher than observed anticorrelation between NH_3_ concentrations and the exchange velocities may be due to the large difference in NH_3_ levels between the synthetic and measured dataset (Fig. 2). Firstly, in source areas primary emissions cause nighttime concentration maxima to occur, whereas exchange rates are highest during late morning hours when PBL growth has diluted the NH_3_ concentrations. CTM modelled data from the grid-cell that includes the Bavarian Forest site (grey lines in Fig. 4d) exhibit a weaker anticorrelation ${r}_{{v}_{{\rm{ex}}},{\chi }_{{\rm{a}}}}$ than the synthetic dataset from the Allgäu region. LOTOS-EUROS’ resolution may explain why it is still somewhat more negative than observed: In each grid cell emissions of NH_3_ take place, causing a slight nighttime maximum. In reality, the stagnant conditions do not allow these emissions to reach a hill site such as the Bavarian Forest measurement tower. Hence, the implicit spatial mixing may explain the stronger anticorrelation found in the measurements.

**Figure 5:**
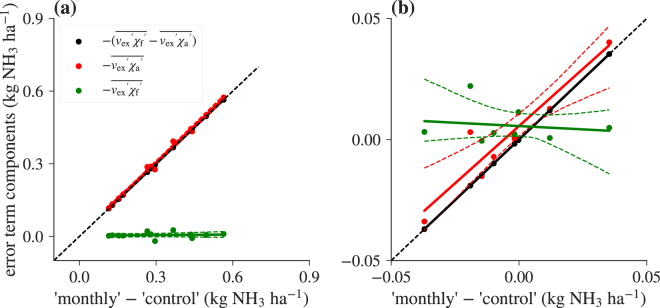
Variation of the individual error terms in equation (9) with the magnitude of the error for (**a**) synthetic and (**b**) measured data. Note that the signs are switched in this graph (deposition is positive) for consistency with Fig. 3.

It is evident from Fig. 5, that, unfortunately for the purposes of correcting biased monthly flux estimates, the known part of the error term ($\overline{{v}_{{\rm{ex}}}\text{'}\cdot {\chi }_{{\rm{f}}}\text{'}}$) contributes much less to the total error than the unknown part ($-\overline{{v}_{{\rm{ex}}}\text{'}\cdot {\chi }_{{\rm{a}}}\text{'}}$). Consequently, the choice between ‘direct’ and ‘monthly’ flux calculation strategies does not substantially change the magnitude of the error. The assumption of a relative error in measured air NH_3_ concentrations appears to be justified from our observations with both modelled and measured concentrations (Fig. 4a,c). However, modelling the correlation of the exchange velocity and air NH_3_ concentrations remains a challenge, as difficulties in the interpretation lead to difficulties in the conceptualisation of an adequate model for ${r}_{{v}_{{\rm{ex}}},{\chi }_{{\rm{a}}}}$ (Fig. 4b,d). Also note that, for NH_3_, both deposition, and emission can occur. We make no distinction between the two in our analysis, as all sites show net deposition on the monthly scale and no artificial management events were modelled. Contrary to deposition velocity models, information about the direction of the flux is removed from the exchange velocity by explicitly separating it from the compensation point in the derivations. Equation (12) appears to work acceptably well for modelling ${r}_{{v}_{{\rm{ex}}},{\chi }_{{\rm{a}}}}$ in the synthetic dataset, but not very well for the measured one. A better course of action than the one presented here might, for example, be based on a multivariate regression using more than one environmental driving factor. However, many potential candidate variables are highly correlated, and the number of parameters of such a multivariate model may quickly approach the number of data points, leading to an increased risk of overfitting and questionable predictive value. We have investigated the potential of fitting the correction factors on a smaller timescale than the averaging period, thereby increasing the number of data points for the regression, but this has been rather unsuccessful in terms of reducing uncertainty. With simple regression approaches, an adequate correction function will certainly be site-specific, and it will not be universally valid for different parameterisations of biosphere-atmosphere exchange schemes. With the increasing availability of optical high-frequency NH_3_ measurement instruments, fitting ecosystem-type and environmental condition specific multivariate correction functions, thereby potentially eliminating the need for site-specific parallel measurements, is a promising outlook, but we assume that the number of NH_3_ concentration measurements currently available is simply too low for this task. However, truly site-independent correction functions that can be readily applied in existing modelling schemes may not even be possible to derive, as they likely depend on a multitude of factors which are not routinely measured. The relationship between *v*_ex_ and *χ*_a_ may be vastly different depending on, for example, the N status of the ecosystem of interest, atmospheric composition, and even the measurement period. In agricultural ecosystems, for instance, there are times when the concentration is largely driven by emission fluxes from the surface, and times when the ambient concentration will drive the flux. The same can be the case for forests before and after leaf-fall^36^. Further research is necessary to develop an optimal strategy to handle these challenges.

Readers should be aware that the observations and derivations made in this study are strictly only valid for a model parameterisation where both *v*_ex_ and *χ*_f_ are not directly dependent on high-frequency observations of the air NH_3_ concentration, *χ*_a_, such as the parameterisation of Massad *et al*.^7^. For instance, Wichink Kruit *et al*.^8^ used an air NH_3_ concentration dependent formulation for the cuticular compensation point to approximate saturation effects within leaf surface water layers. In this case, it would be advisable to use a corrected formulation based on the ‘direct’ variant, so that one *χ*_a_-dependent covariance in the correction term can be eliminated. Other than that, all derivations demonstrated here remain the same, should be adaptable to other parameterisations and model structures in a straightforward manner, and they are valid for any arbitrary averaging period.

## Conclusions

We have demonstrated and formally shown that commonly used averaging strategies for the prediction of long-term average fluxes from long-term average measurements of NH_3_ concentrations (e.g., from denuder or passive sampler records) and high-frequency micrometeorology are biased. The magnitude and variation of this bias is dependent on the biosphere-atmosphere-exchange scheme used, and measurement site characteristics, such as surface, parameters, pollution level and the distance to NH_3_ sources. The magnitude of errors in predicted fluxes introduced by using uncorrected averaging schemes is directly proportional to the (anti-)correlation of NH_3_ exchange velocities and ambient concentrations, which is expected to be significant due to saturation effects on wet leaf surfaces^37–39^, deposition history-dependent compensation points^7,8^, and their inherent dependence on the same environmental variables. Relative errors of up to 100% deviation from ‘control’ and higher were observed in the synthetic dataset, whereas measured data showed both over- and underestimations of less than 25% that compensated each other over the course of the measurement period. The proposed correction scheme consists of

1. Measuring time-series of average NH_3_ concentrations with low-frequency, low-cost monitoring equipment,
2. Measuring meteorological drivers at a high-frequency with standard instrumentation,
3. Taking parallel measurements with a high-frequency NH_3_ monitor for a limited time to parameterise functions to estimate the standard deviation of NH_3_ concentrations (equation (11)), and the correlation of air NH_3_ concentrations with the exchange velocity (equation (12)),
4. Calculating corrected monthly average fluxes using equation (13).

The results of our first tests appear promising, but uncertainties in estimating aforementioned correlation have to be overcome in the future. In its current state, low-frequency concentration measurements need to be accompanied by high-frequency measurements for a certain (yet to be determined) amount of time to derive valid site-specific correction functions. In-depth model structure analyses and multi-site studies, especially at those with higher NH_3_ concentrations and possibly emission fluxes, may give further valuable insight into the exact mechanics behind the dominant source of the error: the correlation of the NH_3_ exchange velocities and air NH_3_ concentrations.

## Electronic supplementary material

Supplementary Material
