# Supplementary material for: The hidden cost of using low-resolution concentration data in the estimation of NH3 dry deposition fluxes
Source: Sci Rep. 2018 Jan 17;8:969. doi: 10.1038/s41598-017-18021-6 (PMC5772562; doi:10.1038/s41598-017-18021-6)
Supplement: Supplementary file 1 — Supplementary Material [file 41598_2017_18021_MOESM1_ESM.pdf]

Supplementary Material to ‘The hidden cost of using low-resolution concentration data in the estimation of NH_3 dry deposition fluxes’

Frederik Schrader[1,*], Martijn Schaap[2], Undine Zöll[1], Richard Kranenburg[2], and Christian Brümmer[1]

[1]Thünen Institute of Climate-Smart Agriculture, Braunschweig, DE-38116, Germany
[2]TNO, Department of Climate, Air and Sustainability, Utrecht, NL-3584, The Netherlands
[*]frederik.schrader@thuenen.de

Additional case studies

Synthetic data

Further evaluations of the correction scheme were carried out for synthetic (LOTOS-EUROS modelled) data from four different grid cells in southern Germany with varying NH_3 concentration levels (Tab. S1), and for five different land-use types (solid lines in Fig. S1). Additionally, we tested splitting the 1-year records into half, using the first half of the year (January to June) to derive parameters for the correction and predicting fluxes for the second half of the year (July to December) as a simple means of validating the method (dashed lines in Fig. S1). Both variants lead to a strong improvement in the average accuracy of the predicted fluxes at most sites, with the exception of the Forst Rotenfels site with arable land. Note that $n \neq 12$ for some sites where the leaf area index is assumed to be zero during certain times of the year (e.g. for arable land).

The land-use scenarios shown here are not necessarily representative for a significant fraction of land-use classes present in the grid cells. They are simply used to illustrate the effects of (not) correcting monthly average fluxes under different conditions.

Site	Latitude	Longitude	χ_a ($\mu g\,NH_3\,m^{-3}$)	T (°C)	ΣP (mm)
Isny	47°41′34.80″ N	10°2′6.00″ E	5.6 ± 5.1 (0.1 to 60.6)	8.1 ± 7.5 (−18.3 to 28.1)	1690
Forst Rotenfels	48°48′50.76″ N	8°23′47.40″ E	1.1 ± 1.3 (0.0 to 11.1)	10.1 ± 7.5 (−12.3 to 31.6)	986
Forst Welzheim	48°52′50.88″ N	9°34′47.64″ E	2.9 ± 2.8 (0.0 to 24.0)	9.7 ± 7.8 (−16.3 to 31.8)	990
Forellenbach	48°56′51.40″ N	13°25′14.05″ E	2.0 ± 2.3 (0.0 to 25.9)	6.0 ± 7.9 (−17.6 to 26.3)	1085

Table S1. Site characteristics of the additionally tested four synthetic datasets in southern Germany. ‘Isny’ corresponds to the synthetic dataset discussed in the main manuscript. Concentration and temperature values are annual averages ± standard deviations with min. and max. values in parentheses. ΣP is total annual precipitation.

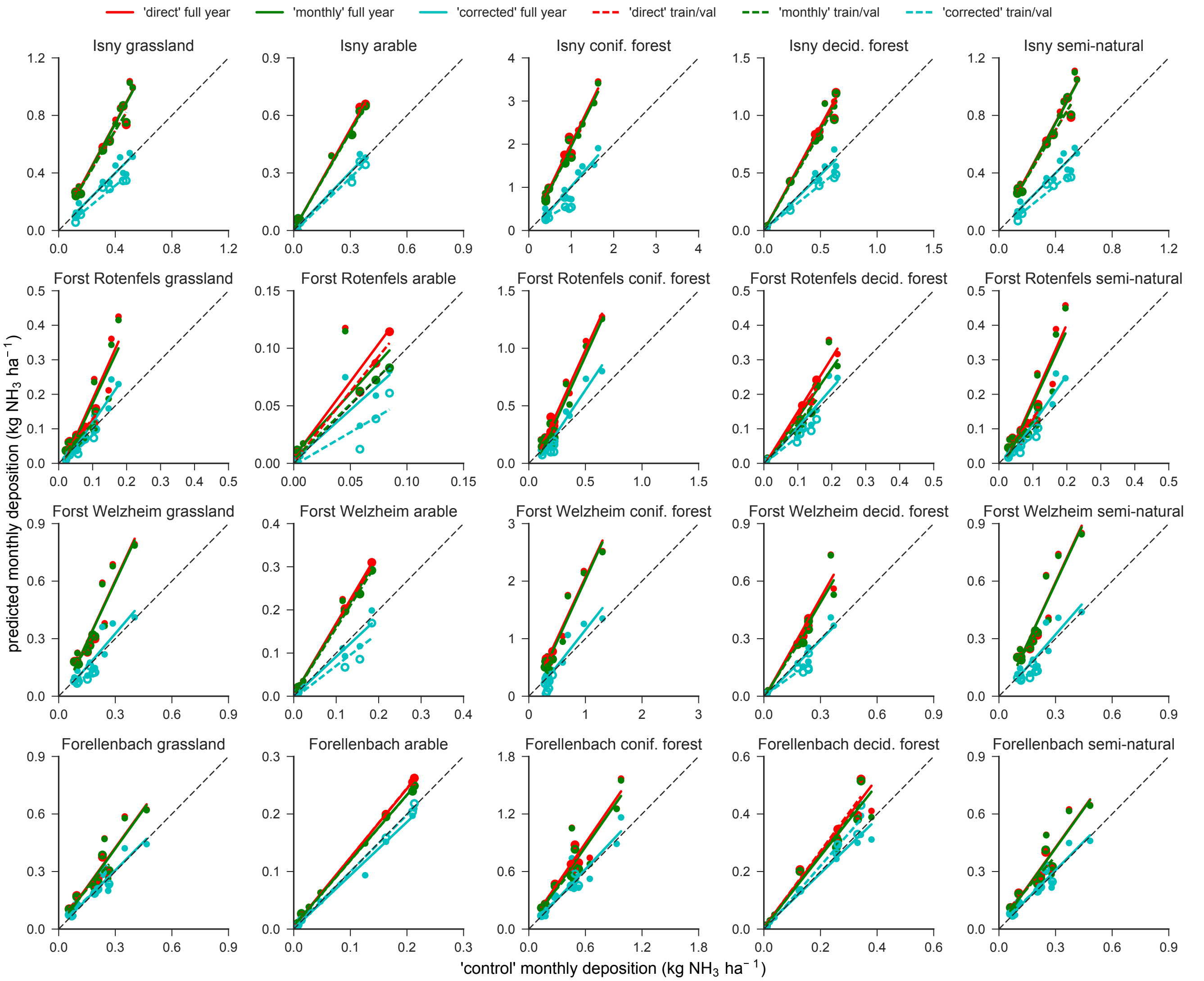

Figure S1. Similar to Fig. 3b,d, for four different sites and five different land-use types. Solid circles and regression lines use the whole modelled year 2016 for derivation of correction factors; hollow circles and dashed lines use the first half of the year for parameter estimation and the second half of the year for validation (data points and regression line only shown for second half). Confidence intervals for the regression are not shown to ensure visual clarity.

Measured data

A similar analysis was carried out for NH_3 concentrations measured at a moorland site in southern Scotland[1] during the years 1995, 1996 and 1998. Mean temperature at the site was 9.1 °C averaged across the sampling periods, and the mean NH_3 concentration was $0.8\,\mu g\,NH_3\,m^{-3}$, with a maximum of $32.9\,\mu g\,NH_3\,m^{-3}$; 95 % of all samples were below $2.9\,\mu g\,NH_3\,m^{-3}$. Concentration measurements and meteorological data were available at a frequency of 30 minutes. The evaluation of the proposed correction scheme was carried out using both the whole measurement period and only the year 1995 as the training dataset for the estimation of σ_{χ_a} and r_{vex,χ_a}.

Results are shown in Fig. S2. Relative errors of up to 63 % in individual months could clearly be reduced using the proposed correction method with a maximum error of 38 % in one month of the 'corrected' variant and below 20 % otherwise. In fact, total errors were reduced from $296.0\,g\,NH_3\,ha^{-1}$ and $203.3\,g\,NH_3\,ha^{-1}$ in the 'direct' and 'monthly' variants, respectively, to a mere $2.6\,g\,NH_3\,ha^{-1}$ after correction ($44.9\,g\,NH_3\,ha^{-1}$ when only using data from 1995 for fitting the correction functions), at a total predicted 'control' deposition of $1625.0\,g\,NH_3\,ha^{-1}$. Note that this prediction is very low compared to measured fluxes at this site ($2.5\,kg\,NH_3-N\,ha^{-1}\,a^{-1} \approx 3.0\,kg\,NH_3\,ha^{-1}\,a^{-1}$)[2], likely due to a too high default minimum external leaf surface resistance in the model parameterisation we used[3,4]. Nevertheless, we have no reason to assume that the performance of the correction scheme would be significantly different after calibrating the biosphere-atmosphere exchange scheme to site-specific conditions.

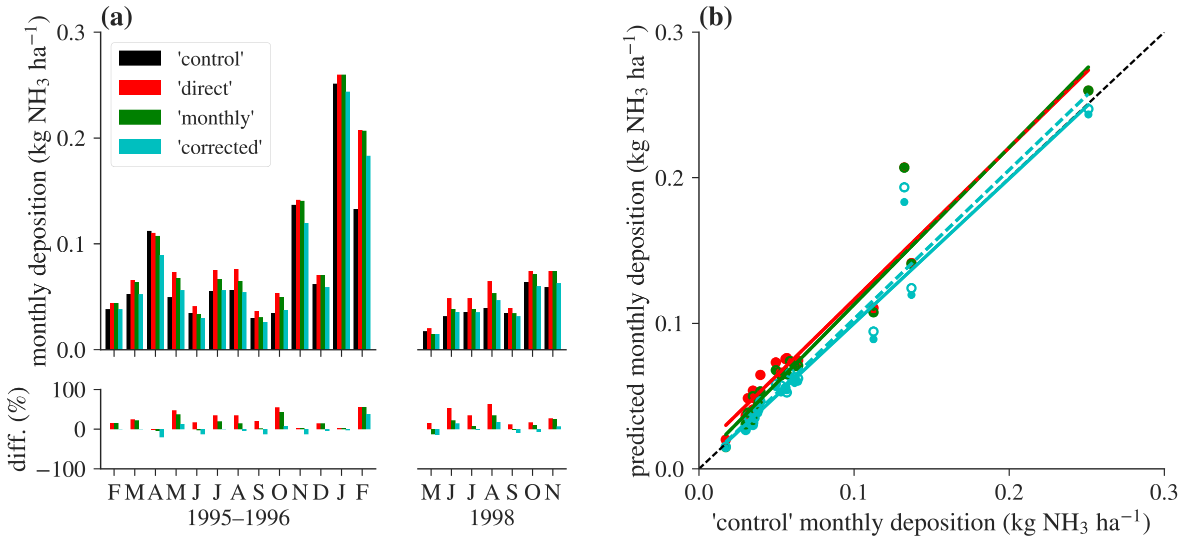

Figure S2. Similar to Fig. 3 for a Scottish moorland site. (a) Predicted cumulative monthly NH_3 deposition for the four scenarios 'control', 'direct', 'monthly', and 'corrected'. Differences are given as percent deviation from 'control'. (b) Predicted cumulative monthly NH_3 deposition of 'direct', 'monthly', and 'corrected' variants against 'control'. Solid circles and regression lines use the whole measurement period 1995–1998 for derivation of correction factors; hollow circles and dashed line use only the year 1995 for parameter estimation. Confidence intervals for the regression are not shown to ensure visual clarity.

Sensitivity to the sampling rate

Although many monitoring networks use a monthly sampling scheme (e.g., MAN in the Netherlands[5]), it is nevertheless instructive to analyse the effect of modelling NH_3 fluxes using average concentration measurements in conjunction with high-frequency meteorological drivers at other temporal resolutions. For example, Hurkuck *et al.*[6] and Dämmgen[7] used weekly measurements obtained with KAPS denuders[8] to model atmospheric nitrogen deposition. We have therefore performed the same analysis as shown in Fig. S2 for averaging times between 1 hour and four weeks for the same dataset. We then evaluated the total relative difference from the 'control' variant in two different ways: (i) as in the main manuscript, by upscaling average fluxes to total data coverage, i.e., multiplying the daily average flux density with the averaging interval, and (ii) without upscaling, i.e., multiplying the daily average flux density with the (fractional) number of days with valid measurements within the averaging interval. The practical difference and reason for performing the analysis in both ways lies in the fact that with

upscaling, the ‘control’ total predicted flux is not equal between different averaging times as soon as there are gaps in the time series that are larger than the averaging interval, whereas without upscaling it is always equal.

As expected, the errors arising from both uncorrected variants (‘direct’ and ‘monthly’) are lowest at the smallest hypothetical sampling intervals (Fig. S3). At the same time, as fewer samples are used to determine $r_{\mathrm{v_{ex}},\chi_a}$, noise increases and the effect of using the proposed correction scheme can actually be worse than not using a correction at all when the averaging period is small. However, this is quickly reversed as soon as concentrations are sampled at a temporal resolution of around half a week or less frequent. As averaging intervals become longer, ‘corrected’ flux predictions fluctuate around ‘control’ (i.e., zero relative error), with occasional outliers, whereas the errors in the uncorrected variants still increase. An interesting lesson to learn from this exercise is that the errors appear to slowly approach a plateau for this dataset. We expect this to be the case for other sites as well, as with increasing averaging time $\overline{\chi_a}$ within each sampling interval converges to the ‘long-term average’ NH_3 concentration at the site, therefore mitigating the effects of spreading out sampling times even longer. In other words, reducing the sampling frequency from, e.g,. monthly to quarterly will have less negative consequences than weekly to monthly.

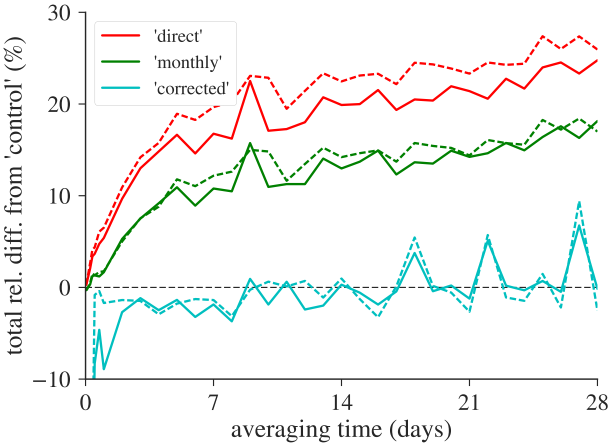

Figure S3. Sensitivity of errors to an increasing sampling interval, exemplarily calculated for the Scottish moorland site previously shown in Fig. S2. Solid lines are with, dashed lines without upscaling to 100 % data coverage within the interval (refer to the text for detailed explanation). The errors are relative to the ‘control’ total predicted flux over the whole measurement period 1995–1998.

References

1. Flechard, C. R. & Fowler, D. Atmospheric ammonia at a moorland site. I: The meteorological control of ambient ammonia concentrations and the influence of local sources. *Q. J. Royal Meteorol. Soc.* **124**, 733–757 (1998). DOI 10.1002/qj.49712454705.
2. Flechard, C. R. & Fowler, D. Atmospheric ammonia at a moorland site. II: Long-term surface-atmosphere micrometeorological flux measurements. *Q. J. Royal Meteorol. Soc.* **124**, 759–791 (1998). DOI 10.1002/qj.49712454705.
3. Massad, R.-S., Nemitz, E. & Sutton, M. A. Review and parameterisation of bi-directional ammonia exchange between vegetation and the atmosphere. *Atmospheric Chem. Phys.* **10**, 10359–10386 (2010). DOI 10.5194/acp-10-10359-2010.
4. Schrader, F. *et al.* Non-stomatal exchange in ammonia dry deposition models: Comparison of two state-of-the-art approaches. *Atmospheric Chem. Phys.* **16**, 13417–13430 (2016). DOI 10.5194/acp-16-13417-2016.
5. Lolkema, D. E. *et al.* The Measuring Ammonia in Nature (MAN) network in the Netherlands. *Biogeosciences* **12**, 5133–5142 (2015). DOI 10.5194/bg-12-5133-2015.
6. Hurkuck, M. *et al.* Determination of atmospheric nitrogen deposition to a semi-natural peat bog site in an intensively managed agricultural landscape. *Atmospheric Environ.* **97**, 296–309 (2014). DOI 10.1016/j.atmosenv.2014.08.034.
7. Dämmgen, U. Atmospheric nitrogen dynamics in Hesse, Germany: Creating the data base 2. atmospheric concentrations of ammonia, its reaction partners and products at Linden. *Landbauforschung Völkenrode* **57**, 157–170 (2007).
8. Peake, E. & Legge, A. H. Evaluation of methods used to collect air quality data at remote and rural sites in Alberta, Canada. In *Proc. 1987 EPA/APCA Symposium on Measurements of Toxic and Related Air Pollutants* (APCA, 1987).
